# Differences in child and adolescent exposure to unhealthy food and beverage advertising on television in a self-regulatory environment

**DOI:** 10.1186/s12889-023-15027-w

**Published:** 2023-03-23

**Authors:** Monique Potvin Kent, Julia Soares Guimaraes, Meghan Pritchard, Lauren Remedios, Elise Pauzé, Mary L’Abbé, Christine Mulligan, Laura Vergeer, Madyson Weippert

**Affiliations:** 1 School of Epidemiology and Public Health, Faculty of Medicine, University of Ottawa, 600 Peter Morand Crescent, Ottawa, Ontario K1G 5Z3 Canada; 2 School of Interdisciplinary Health Sciences, Faculty of Health Sciences, University of Ottawa, Ottawa, Canada; 3 Department of Nutritional Sciences, Faculty of Medicine, University of Toronto, Toronto, Canada

**Keywords:** Food marketing, Television, Children, Adolescents

## Abstract

**Background:**

Food and beverage promotion is a contributor to children’s dietary behaviours, and ultimately, downstream health consequences. Broadcast television remains an important source of such advertising. The objective of this study was to examine and compare children and adolescent’s exposure to food advertising on television in Canada over an entire year in a self-regulatory environment.

**Methods:**

Television advertising data for 57 selected food and beverage categories were licensed from Numerator for 36 stations in Toronto, for 2019. The estimated average number of advertisements viewed by children aged 2–11 and adolescents aged 12–17 was determined overall, by food category, and by marketing technique. The healthfulness of advertisements was also assessed using Health Canada’s Nutrient Profile Model.

**Results:**

Overall in 2019, children viewed 2234.4 food ads/person/yr while adolescents viewed 1631.7 ads, exposure for both groups stemmed primarily from stations with general appeal, and both age groups were exposed to a range of powerful marketing techniques. Exposure to advertising for restaurants, snacks, breakfast food and candy and chocolate was high among both age groups and the healthfulness of most advertised products was considered poor. Adolescents were exposed to 36.4% more food products classified as unhealthy, had higher exposure to all marketing techniques examined, and were exposed to substantially more child-related marketing techniques compared to children.

**Conclusion:**

Children and adolescents were heavily exposed to food advertisements on television in 2019. Despite current self-regulatory policies, children’s exposure to unhealthy food and beverages remains high. Differences in exposure to food advertisements by food category and healthfulness may suggest that adolescents are being disproportionately targeted by food companies as a result of self-regulatory marketing restrictions.

**Supplementary Information:**

The online version contains supplementary material available at 10.1186/s12889-023-15027-w.

## Introduction

Obesity among youth is an issue worldwide [1–3]. In Canada, obesity in children and adolescents has increased significantly over time, and data from 2015 indicate that 10.4% of children aged 5 to 11 years and 13.8% of adolescents aged 12–17 years have obesity [4]. Poor dietary intake contributes to obesity and youth diets are characterised by high levels of ultra-processed foods and beverages that are elevated in added fats, sugar, and salt [5]. Evidence from systematic reviews has demonstrated that exposure to the marketing of unhealthy food and beverage products can contribute to obesity and other chronic diseases associated with poor diet by influencing food preferences and food intake [6, 7].

Although children and adolescents are spending increasing amounts of their leisure time on digital devices, broadcast television remains an important source of food marketing exposure among young people [8, 9]. According to 2017/18 data, children in Canada spend an average of 17.3. hours/week watching television, while adolescents spend 13.9 hours/week viewing this media [10]. Much of the literature to date on food advertising on television has focused on school-aged children. One study involving 22 countries demonstrated that advertisements containing nutrient-poor foods were more common during popular children’s viewing times, and that advertisements for these foods frequently contained persuasive and child-directed messaging [11, 12]. This is especially problematic since children lack the cognitive ability to comprehend persuasive marketing techniques [13–15]. Indeed, research shows that the persuasive techniques used in food and beverage advertising to children can alter attitudes, preferences, and food consumption which can ultimately result in poorer health outcomes [7, 16].

Adolescents are also a key target market for the food and beverage industry as they are more independent and have greater purchasing power than children [17]. Neurocognitively, they are also vulnerable due to their overactive reward pathways, poor impulse control as well as heightened peer influence [18]. A recent study found that Canadian television stations aired 3.3 food advertisements per hour on programs designated by broadcasters as targeted at adolescents (ages 12–17) compared to 1.5 food advertisements per hour on programs targeted at children (ages 2–11) [8]. Another recent study examining Canadian adolescent exposure to food and beverage advertising found that despite the decreases in exposure between May 2011 and 2016, adolescents aged 12–17 living in Toronto were nonetheless exposed to over 150 food and beverage advertisements on television in May 2016 [19].

In light of the evidence base on the harmful effects of food marketing, countries such as the UK, Mexico, and Chile have introduced government policies to protect children from unhealthy television food advertising [20]. With the exception of the UK, few countries have extended these protections to adolescents despite their vulnerability to unhealthy food advertising. Other countries such as the United States, Australia, and Canada (with the exception of the province of Quebec) have taken a self-regulatory approach to restricting food advertising and here again, adolescents have been excluded from protections [21, 22]. In Canada, advertising to children under age 12 is self-regulated and 15 food companies have committed to restricting unhealthy food advertising to children in a variety of media and settings through the *Children’s Food and Beverage Advertising Initiative* [18]. Though this initiative has been shown to be ineffective at protecting children, much of the research evaluating this initiative has been conducted using television data collected over a one-month period [12, 23]. Research on adolescent exposure to food advertising is also very limited and based on monthly data [23]. Little is known about whether the current self-regulatory policy confers any protection to adolescents or whether, in the alternative, adolescents are exposed to higher levels of food advertising given that the food and beverage industry has not committed to protecting this age group.

Given this critical gap in data, comparing child and adolescent exposure to food advertising is essential to inform food marketing restrictions. A new bill to restrict food marketing to children was introduced in the Canadian House of Commons in February 2022, Bill C-252, and once again, adolescents have been overlooked [24]. The purpose of the current study was to determine whether there are differences in child and adolescent exposure to food and beverage advertising on television and to marketing techniques used in food advertising. It was hypothesized that children would be exposed to fewer food and beverage advertisements compared to adolescents.

## Materials and methods

### Data sample

Data on television viewership and advertisements airing from January to December 2019 were licensed from Numerator, a marketing information and advertising intelligence company. The advertising data included 57 selected food categories broadcast on 36 television stations in Toronto. The 57 food categories were selected from 112 possible categories because they were either known to be advertised heavily to children and adolescents [11] and/or because of their contribution to children’s food intake and diet quality, including both less healthy and healthier product categories. These food categories were then aggregated into 13 food categories which included bread; sweet baked goods/desserts; candy and chocolate; breakfast food; dairy; condiments; entrees and meat (including fish, poultry, and meat products); fruit and vegetables; beverages (excluding milk and water); miscellaneous; snacks; water; and restaurants. See Additional file 1 for a detailed breakdown of all food categories and their definitions. Toronto was selected as it is the largest media market in Canada and has the largest panel size of children aged 2–11 years (*n* = 175) and adolescents aged 12–17 years (*n* = 106).

Television audience viewership data are provided by Numerator though these data are collected by Numeris, an organization that maintains a panel and collects audience viewership data from a stratified random sample of households proportional to the population. Each person on Numeris’ panel wears a portable device which captures the stations to which the television set is tuned when each panel member is near the television. Data are then weighted according to demographic variables including age, sex, and household size. This enables the examination of advertising viewership by age group (e.g., children aged 2–11 years and adolescents aged 12–17 years).

All stations captured for the Toronto market (*n* = 36) were examined and all 24 hours of television per day were analyzed.

### Frequency of food and beverage advertisements

The frequency of food and beverage advertisements was extracted from AdQuest, an advertising platform licensed from Numerator. These data included the number of unique advertisements and the weighted frequency of advertisements. An advertisement was considered unique if it differed from others in terms of content, language, or duration. To calculate the weighted frequency, the number of products in an advertisement was multiplied by the number of times an advertisement was broadcasted. For example, if there were 2 products in an advertisement broadcasted 500 times, the weighted frequency would be 1000. If the number of unique products exceeded 3, then it was only counted as having 3 products (i.e., 4 products in an advertisement broadcasted 500 times would have a weighted frequency of 1500). The limit was capped at 3 products to remain consistent with Numerator methodology.

### Exposure to food and beverage advertisements

Numerator expresses exposure as “ratings”, which is the approximate percentage of a population that has viewed an advertisement. Ratings summed across a defined period of time (in this case 24-hour programming broadcast from January to December 2019) are known as gross rating points (GRPs). GRPs are calculated by dividing the total impressions by the total population of the media market and multiplying by 100 (Impressions / Population × 100). GRPs were determined by age group and were divided by 100 to calculate the estimated average exposure to food and beverage advertising, overall or by food category. GRPs will hereinafter be referred to as “exposure”, however note that this measure is approximate and not an absolute measure of child and adolescent exposure.

### Content analysis of food marketing techniques

A content analysis of all advertisements aired on the captured stations in 2019 in Toronto was conducted. Excluding duplicates and data that was missing due to technical problems extracting advertisements from the AdQuest platform (*n* = 22), a total of 1365 unique ads were coded (total frequency = 1,670,912, 97.1% of total food advertising frequency). Marketing techniques as described in Table 1 were recorded as present or absent and were counted once per ad, regardless of how many products were featured. The advertisements were analyzed by three trained research assistants using a previously developed coding manual [25]. During training, interrater reliability of 0.93 was calculated based on practice samples. Advertisements were then coded by randomly distributing unique advertisements among the three coders, and a researcher oversaw the data looking for any inconsistencies, which were settled via consensus.

**Table 1:** Marketing techniques examined

Marketing technique	Description
Child actors	Main characters in the advertisement are children (0–12 years), or have childlike voices
Child products	A product that appeals to children due to the type/nature of product (e.g., candy, compartment snacks), its shape, colour and/or design
Child-appealing characters	Cartoon characters, animals, or imaginary, fantasy, or virtual creatures
Child language	The level of language is that commonly used by children or language is directed at children (e.g., “Hey Kids”)
Child-appealing special effects	Lettering, colours, special effects, animation, music, songs or jingles that appeal to children
Child themes	Child-appealing themes linked to fantasy, magic, mystery, suspense, adventure, or virtual worlds are featured
Use of spokes-characters	E.g., brand-owned characters such as Tony the Tiger
Parent-child situations	Situations that play on the parent-child relationship or other authority-based relationship (i.e., coach-child or teacher-child)
Use of licensed characters	E.g., Dora the Explorer or Spiderman
Cross-promotions	Cross-promotions to movies or television shows watched by children
Child incentives	Free gifts including toys, books, collectibles aimed at children
Teen actors	Youth (12–17 years) were prominently featured
Teen language	E.g., “hey dude”
Teen music	E.g., rap
Teen themes	Themes based on adolescent activities or interests (e.g., socializing, school-related activities like dances, sports or extreme sports/risk-taking behavior, adolescent-directed humor, freedom, popular music/culture, video games)
Teen incentives	E.g., gift card to movie theatre
Teen humour	E.g., boy wiping out on a skateboard
Contest/sweepstakes	Prizes are given away at no charge to the participants and there is a competition (not every consumer will win).
Celebrity endorsements	E.g., musical groups, film stars, athletes, etc.
Health claims	Health or nutrition claims
Price promotions	Price related premiums or rebates (e.g., bonus offers, calls to action to encourage purchase)
Call to action - online	Sending viewers online to access brand website, app, etc.

### Nutritional analysis

The nutritional information for each product featured in an advertisement was primarily collected using the 2017 Food Label Information Program (FLIP), a large database containing food label information for over 17,000 Canadian products from three grocery retailers (Metro, Sobeys, and Loblaws) [26]. For restaurant and fast-food items, the 2016 Menu-FLIP with over 12,000 restaurant food items was used [27]. If products were not in FLIP or Menu-FLIP, the nutrition information was obtained from 1) the company’s Canadian website, 2) the product’s Nutrition Facts table found online, or 3) the company’s American website, or 4) a similar product from the Canadian Nutrient File was substituted if the original product could not be found.

Each advertisement was further classified as “unhealthy” or “healthy” based on the Nutrient Profile Model (NPM) by Health Canada designed to identify products that should not be marketing to children [28]. This classification is based on nutrient thresholds for added fat, sugar and salt. If any product with added fat, sodium and/or sugar within advertisements exceeded the thresholds, then the entire advertisement was deemed “unhealthy”. See Additional file 2 for the detailed criteria outlined by Health Canada. Note that only products were included in the analysis; data were not collected for brand advertisements (e.g., advertisement featuring no identifiable food products).

### Data analysis

The frequency of advertisements and exposure to advertisements were determined by media market, age group, and by food category. The frequencies of marketing techniques and proportion of advertisements that were “unhealthy” were calculated. The relative and absolute differences between children and adolescent’s exposure to food and beverage advertising were also calculated, using adolescents as the comparator group.

## Results

Overall, a total of 1,720,763 food advertisements were broadcast across 36 stations in Toronto in 2019 as shown in Table 2. Each child viewed 2234.4 food advertisements on these stations in 2019, while each adolescent in Toronto viewed 1631.7 food advertisements over the entire year. In relative and absolute terms, adolescents viewed 27.0% less or 602.7 fewer advertisements in 2019 compared to children. Children’s greatest exposure was on Citytv (257.4 ads/person/year), YTV (198.8 ads/person/year), CTV (198.3 ads/person/year), SportsNet Ontario (176.8 ads/person/year), and Global (140.8 ads/person/year.) Adolescents’ greatest exposure was from CTV (142.1 ads/person/year), YTV (133.0 ads/person/year), Citytv (125.0 ads/person/year), Global (112.7 ads/person/year), and TSN4 (94.3 ads/person/year).

**Table 2:** Exposure to food products advertised on 36 stations in Toronto in 2019

Stations	Freq (%)	Ads/person/year			
		Children Ads/person/yr	Adolescents Ads/person/yr	Absolute difference Children vs adolescents	Relative difference %
CBC	42,672 (2.5)	64.3	46.8	−17.5	−27.2%
CBC News Network	24,120 (1.4)	7.6	11.3	3.7	48.7%
CHCH	18,756 (1.1)	14.4	12.9	−1.5	−10.4%
Citytv	56,887 (3.3)	257.4	125.0	− 132.4	− 51.4%
CMT	71,022 (4.1)	19.6	20.0	0.4	2.0%
CP24	5229 (0.3)	29.3	26.7	−2.6	−8.9%
CTV	45,295 (2.6)	198.3	142.1	−56.2	−28.3%
CTV 2	47,099 (2.7)	69.6	46.1	−23.5	−33.8%
CTV Comedy Channel	56,703 (3.3)	138.5	80.6	−57.9	− 41.8%
CTV Drama Channel	52,341 (3.0)	20.9	32.8	11.9	56.9%
CTV Life Channel	76,656 (4.5)	16.9	10.0	−6.9	−40.8%
CTV Sci Fi Channel	61,568 (3.6)	84.8	48.5	−36.3	−42.8%
Discovery Channel	45,966 (2.7)	43.8	30.1	−13.7	−31.3%
Disney Channel	26,168 (1.5)	52.3	42.6	−9.7	−18.5%
DTour	59,942 (3.5)	6.3	18.5	12.2	193.7%
Food Network	71,387 (4.1)	77.1	70.3	−6.8	−8.8%
Global	53,986 (3.1)	140.8	112.7	−28.1	−20.0%
HGTV	37,133 (2.2)	69.2	40.4	−28.8	−41.6%
History	50,800 (3.0)	30.1	35.6	5.5	18.3%
Investigation Discovery	14,677 (0.9)	2.5	10.2	7.7	308.0%
MTV	72,599 (4.2)	35.2	25.7	−9.5	−27.0%
Much	84,728 (4.9)	47.0	63.1	16.1	34.3%
National Geographic	13,592 (0.8)	4.3	5.8	1.5	34.9%
OLN	70,146 (4.1)	12.8	21.7	8.9	69.5%
Omni	50,466 (2.9)	2.0	0.5	−1.5	−75.0%
Showcase	61,333 (3.6)	69.0	47.2	−21.8	−31.6%
Slice	59,398 (3.5)	12.0	21.6	9.6	80.0%
Sportsnet 360	58,678 (3.4)	19.2	17.9	−1.3	−6.8%
SportsNet Ontario	49,654 (2.9)	176.8	89.3	−87.5	− 49.5%
Teletoon	67,984 (4.0)	84.8	43.0	−41.8	−49.3%
The Weather Network	25,117 (1.5)	3.7	8.4	4.7	127.0%
TSN2	11,405 (0.7)	4.9	3.2	−1.7	−34.7%
TSN4	45,981 (2.7)	157.1	94.3	− 62.8	−40.0%
VisionTV	11,061 (0.6)	1.8	3.2	1.4	77.8%
W Network	52,594 (3.1)	61.6	90.4	28.8	46.8%
YTV	67,530 (3.9)	198.8	133.0	−65.8	−33.1%
**Total**	1,720,673 (100.0)	2234.4	1631.7	−602.7	−27.0%
**Average by station**	47,796	62.1	45.3	−16.7	−27.0%

Source: Numerator, 2019. Analysis based on the 57 selected food categories

### Differences in children and adolescent’s exposure to food advertising by food category

The most frequently advertised food categories in 2019 were restaurants (49.1% of advertisements), snacks (9%), candy and chocolate (9%), and dairy (8.4%) (Table 3). Both children and adolescents were most exposed to advertisements for restaurants (1145.5 and 813.6 ads/person/year, respectively) and snacks (204.2 and 149.6 ads/person/year respectively). Additionally, children were also highly exposed to advertising for breakfast food (188.3 ads/person/year), candy and chocolate (161.6 ads/person/year) and beverages 117.5 ads/person/year) while adolescents were highly exposed to dairy (136.4 ads/person/year), breakfast food (132.4 ads/person/year) and candy and chocolate (122.6 ads/person/year). Child and adolescent exposure to other categories, such as water, fruits and vegetables, and bread were markedly lower. Adolescents were exposed to less advertisements compared to children across all food categories; in relative terms this was most notable for restaurants (− 29%), fruits and vegetables (− 31.8%), and breakfast food (− 29.7%). In absolute terms, the greatest negative differences between children and adolescent’s exposure were for restaurants (− 331.9 ads/child), snacks (− 54.6 ads/child), and breakfast food (− 55.9 ads/child).

**Table 3:** Exposure to food products advertised on 36 stations in Toronto by food category in 2019

Food category	Freq (%)	Ads/person/year			
		Children Ads/ person /yr	Adolescents Ads/ person /yr	Absolute difference Children vs adolescents	Relative difference %
Bread	12,485 (0.7)	17.4	13.7	−3.7	−21.3%
Sweet baked goods/desserts	61,723 (3.6)	59.8	44.6	−15.2	−25.4%
Candy and chocolate	154,970 (9.0)	161.6	122.6	−39.0	−24.1%
Breakfast food	105,882 (6.2)	188.3	132.4	−55.9	−29.7%
Dairy	145,342 (8.4)	170.1	136.4	−33.7	−19.8%
Condiments	21,944 (1.3)	39.3	30.9	−8.4	−21.4%
Entrees	59,815 (3.5)	66.5	53.0	−13.5	−20.3%
Fruits/vegetables	26,866 (1.6)	37.7	25.7	−12.0	−31.8%
Beverages	89,143 (5.2)	117.5	87.1	−30.4	−25.9%
Miscellaneous	70,608 (4.1)	69.3	52.6	−16.7	−24.1%
Snacks	155,323 (9.0)	204.2	149.6	−54.6	−26.7%
Water	14,346 (0.8)	13.3	10.8	−2.5	−18.8%
Restaurants	844,224 (49.1)	1145.5	813.6	−331.9	−29.0%
**Total**	**1,720,673 (100.0)**	**2234.4**	**1631.7**	**−602.7**	**−27.0%**

Source: Numerator, 2019. Analysis based on the 57 selected food categories

### Differences in children and adolescent’s exposure to marketing techniques

Children and adolescents’ exposure to examined marketing techniques is presented in Table 4. Overall, calls to action (34.7% of all advertisements), health appeals (32.3%), and child-appealing products (30.3%) were the most frequently featured marketing techniques over the entirety of 2019 in Toronto. Children and adolescents were heavily exposed to similar marketing techniques, and exposure was highest for both age groups to calls to action (547.1 and 764 ads/person/year for children and adolescents respectively), health appeals (526.7 and 720.4 ads/person/year for children and adolescents respectively), child-appealing products (486.4 and 664.3 ads/person/year for children and adolescents respectively), and child-appealing special effects (432.1 and 586.6 ads/person/year for children and adolescents respectively). Adolescents had higher exposure to all marketing techniques examined compared to their younger counterparts. The greatest relative differences in exposure were for adolescent humour (+ 44.7%), adolescent music (+ 42%), and adolescent language (+ 44.4%) while the greatest absolute differences were for call to action (+ 216.9 ads/child), health appeal (+ 193.7 ads/child), and child-appealing product (+ 177.9 ads/child).

**Table 4:** Exposure to food products advertised on 36 stations in Toronto by marketing techniques in 2019

Marketing techniques	Freq (%)	Ads/person/year			
		Children Ads/ person /yr	Adolescents Ads/ person /yr	Absolute difference Children vs. adolescents	Relative difference %
Child actor	339,343 (20.3)	330.2	442.8	112.6	34.1%
Child-appealing product	506,107 (30.3)	486.4	664.3	177.9	36.6%
Child-appealing characters	344,919 (20.6)	367.5	500.0	132.5	36.1%
Child language	93,567 (5.6)	107.6	150.3	42.7	39.7%
Child-appealing special effects	426,094 (25.5)	432.1	586.6	154.5	35.8%
Child themes	230,821 (13.8)	247.9	335.3	87.4	35.3%
Use of spokes-characters	323,490 (19.4)	339.9	462.8	122.9	36.2%
Use of licensed characters	10,138 (0.6)	9.9	12.9	3.0	30.3%
Cross-promotions	53,760 (3.2)	52.6	72.1	19.5	37.1%
Child incentives	18,615 (1.1)	18.6	24.1	5.5	29.6%
Adolescent actor	229,355 (13.7)	223.7	302.0	78.3	35.0%
Adolescent language	37,575 (2.2)	49.3	71.2	21.9	44.4%
Adolescent music	30,280 (1.8)	43.8	62.2	18.4	42.0%
Adolescent themes	306,385 (18.3)	304.4	415.0	110.6	36.3%
Adolescent incentives	5289 (0.3)	6.9	8.1	1.2	17.4%
Adolescent humour	33,527 (2.0)	43.4	62.8	19.4	44.7%
Contest/sweepstakes	59,618 (3.6)	63.6	81.0	17.4	27.4%
Celebrity endorsement	77,925 (4.7)	83.5	110.5	27.0	32.3%
Parent-child situations	316,086 (18.9)	281.5	371.5	90.0	32.0%
Health appeal	538,910 (32.3)	526.7	720.4	193.7	36.8%
Price promotion	353,754 (21.2)	331.6	461.9	130.3	39.3%
Call to action	579,213 (34.7)	547.1	764.0	216.9	39.6%

Source: Numerator, 2019. Analysis based on the 57 selected food categories

### Differences in children and adolescent’s exposure to food advertising by nutritional content

A greater proportion (91.3%) of the advertisements broadcast in 2019 in Toronto were unhealthy compared to those considered healthy (8.7%) (Table 5). Children’s exposure to food advertisements that were classified as unhealthy was 760 ads/person/year while adolescent exposure was to such advertisements was markedly higher at 1036.7 ads/person/year. Adolescents viewed 36.4% or 276.7 more advertisements per person that were unhealthy compared to children.

**Table 5:** Exposure to food products advertised on 36 stations in Toronto by healthfulness in 2019

NPM classification		Ads/person/year			
	Freq (%)	Children Ads/person/yr	Adolescents Ads/ person /yr	Absolute difference Children vs. adolescents	Relative difference %
Healthy	74,617 (8.7)	64.3	90.4	26.1	40.6%
Unhealthy	783,855 (91.3)	760.0	1036.7	276.7	36.4%

Source: Numerator, 2019. Analysis based on the 57 selected food categories on products where nutritional information was available

## Discussion

Overall, our results indicate that while children in Toronto were exposed to higher levels of food advertising than adolescents and exposed to a myriad of marketing techniques in 2019, adolescents’ exposure to unhealthy food advertising and marketing techniques was more than 30% greater than children.

### Differences in child and adolescent exposure

This study revealed that both children and adolescents were heavily exposed to food advertising on television in Toronto in 2019. Though it was hypothesized that children would be exposed to fewer food and beverage advertisements compared to adolescents, our results showed the opposite. Children aged 2–11 years were exposed to 2234 food advertisements on the examined stations or 6.1 ads per day while adolescents aged 12–17 years viewed 27% fewer ads and were exposed to 1631.7 food advertisements or 4.5 ads per day. This is consistent with past research in Canada that showed higher rates of food advertising in programming targeted at children [8, 29]. Similar trends have been noted in the United States, where a study found that in 2017, adolescents viewed an average of 9.4 ads per day, while children viewed an average of 10 ads per day [30]. The noted exposure differences between age groups may in part, be due to changes in media consumption among adolescents, with less time being spent watching broadcast television among adolescents aged 12 to 17 years compared to children aged 2 to 11 years [10]. Other research in Canada has shown that there was a decline in adolescent exposure to food advertising on television between May 2011 and May 2016 [19]. Given decreased adolescent broadcast television viewership, the food and beverage industry may be re-directing their advertising dollars from television to digital media where teens are omnipresent in order to reach this market [31, 32].

Both children and adolescents were exposed to similar food categories. Restaurants, snack foods, breakfast foods, and candy and chocolate were figured prominently, while exposure to other categories, such as fruits and vegetables or water, was markedly lower. Restaurants (fast food and non-fast food) accounted for the largest source of advertising exposure relative to other food categories for both children and adolescents, with children exposed to 1145.5 advertisements (more than 3 ads/day) while adolescents were exposed to 813.6 advertisements (2.2 ads/day) in 2019. This high level of restaurant advertising is concerning from a public health perspective as experimental research has shown that exposure to fast food advertising induces reward-related neural pathways in adolescents and contributes to an overall greater intake of fast food [33]. Consuming fast food has also been linked with poor health outcomes in children and adolescents including weight gain/obesity, increased risk of cardiovascular disease, difficulties in insulin functioning, and diabetes [34]. Furthermore, research conducted in the UK has indicated that eating at full-service restaurants contributes to excessive caloric intake, with many meals from these types of restaurants exceeding calorie counts of meals from fast food restaurants [35]. High levels of restaurant advertising on television could be stymied by government policy. To date, only one restaurant manufacturer participates in the self-regulatory *Children’s Food and Beverage Advertising Initiative* (CAI) [21]. Government legislation that restricts unhealthy food marketing to children and adolescents would level the playing field here by applying to all food and beverage companies including all fast food and non-fast food restaurants.

Though children were exposed to more food advertising overall, adolescents were exposed to 36.4% more unhealthy food advertisements in 2019. Given that unhealthy food advertising has been causally linked to diet, this is worrisome [36]. Adolescent diets are characterized by high consumption of sugar, fats, and salts and low consumption of fruits and vegetables [37, 38]. Such results beg the question of whether focusing marketing restrictions exclusively on children is adequate when adolescents are clearly being bombarded with unhealthy food advertising on television. Here, the UK has taken the lead as their television advertising restrictions apply to children under the age of 16 [39]. Our results suggest that Canada should seriously consider following this example.

### Child and adolescent exposure to marketing techniques

Advertising impact is a function of both exposure and power (i.e. the design and execution of the advertising message) [40]. Children and adolescents in Toronto were both highly exposed to a broad range of powerful marketing techniques in 2019 though overall, adolescents had higher exposure (> 30%) to all marketing techniques examined compared to their younger counterparts. Most notably, the highest exposure across both age groups was for calls to action, health appeals, the use of child-appealing products, and child-appealing special effects. Advertisements viewed by children and adolescents also commonly featured child and teen actors and themes and child-appealing characters such as cartoons. Children and adolescents viewed 340 and 463 exposures respectively to spokes-characters in television food advertising and 83 and 111 exposures respectively to celebrity endorsements in 2019. Both of these techniques have been shown to be particularly appealing to both children and adolescents [41]. The frequent inclusion of such marketing techniques in television advertisements to children suggests the *Children’s Food and Beverage Advertising Initiative* (CAI) is not effectively preventing the use of most marketing techniques intended to appeal to children and that much remains to be done to reduce children’s exposure to these techniques. The fact that adolescents were exposed to these marketing techniques at a higher level than children suggest they are being heavily targeted. The food marketing literature is evolving to recognize that adolescents’ ability to understand and recognize persuasive appeals may not provide an adequate defense against the harmful impact of unhealthy food advertisements [42]. Again, governments need to consider extending protections to this age group.

### Policy implications

Overall, our results further reinforce previous evidence which indicates that self-regulatory policies are not efficiently reducing children’s exposure to unhealthy food advertising on television or the power of such marketing [40, 43, 44]. The CAI has been previously criticized for its low participation rates, inadequate definition of advertising, high audience thresholds to trigger pledges, and weak nutritional criteria [8, 44]. In the summer of 2021, a new industry-wide voluntary food code, the *Food and Beverage Advertising Code* was announced and is to be implemented by the summer of 2023 [45]. This new code applies to children under age 13 and is a poor imitation of the *Quebec Consumer Protection Act* which restricts all commercial advertising directed to children under the age of 13. In particular, this new industry code excludes many media forms, and does not explicitly outline how child targeting will be determined, provide nutritional criteria for in-store and out of home meals nor include financial penalties for infractions. With the growing body of literature pointing to the ineffectiveness of self-regulation of food marketing to children, Bill C-252 which was recently introduced in the Canadian federal House of Commons, is a more viable option given the breadth of its media coverage in terms of protecting children from unhealthy food marketing across media and settings. This bill would restrict unhealthy food marketing to children under age 13 in a variety of media and child settings as well as restrict the use of marketing techniques commonly used to target children [24]. The results of this study can be used as baseline data to evaluate the effectiveness of the new potential Canadian law and industry code.

The results of this study also suggest a need to include adolescents in food marketing policy efforts. While children are widely recognized as a priority age group for food marketing restrictions, our results point to the insufficiency of regulation solely targeting children [46, 47]. The levels of exposure observed among adolescents in this study represent a significant loophole in current self-regulatory policies and in the new proposed Canadian Bill C-252. As previously described, adolescents face a unique set of conditions given their neurodevelopmental stage, increasing independence and financial resources, and greater susceptibility to peer influence [18, 33]. To compensate for profits lost to food marketing restrictions to children, companies may pivot to target adolescents by designing food advertisements that capitalize on these unique developmental vulnerabilities [48]. The new Bill C-252 proposes a review 5 years post-implementation of the impact of the new legislation on unhealthy food advertising directed at adolescents aged 13–16 years [24]. Continued monitoring of both children and adolescents’ exposure to food advertising on television is warranted to inform future policy efforts in Canada.

## Limitations

Although this is the first study to compare child and adolescent exposure to food marketing and its associated marketing techniques using licensed commercial full-year data across a large number of stations, some limitations should be noted. First, the exposure data from this study can only be considered as a proxy measure since the portable devices used to measure exposure only captured the television stations being broadcasted. As a result, it cannot be stated with certainty that the advertisements were viewed by participants, only that the participants were near their televisions at the time of broadcast. Another limitation is that this study only presents data from one market (Toronto) and though it is the largest media market in Canada, it may not be representative of the entire country. Furthermore, although we were able to compare children and adolescents, other comparisons by demographic characteristics (socioeconomic status, sex, race) were not included, either due to not having sufficient sample sizes (i.e., sex) or simply lack of data availability (i.e., race, socioeconomic status). There were also several key limitations linked with the nutritional data collected in this study. We were unable to collect nutritional information for 51.4% of the advertisements. Particularly products from brand advertisements, sit-down restaurants and new seasonal products that were not included in the FLIP databases and could not be derived from other sources. Sit-down restaurants in particular did not provide complete data (serving sizes or complete nutrient information) for available products on their websites which precluded a nutritional analysis. Nutritional information could also not be collected on brand advertisements as these ads do not feature any food products. Since FLIP and Menu FLIP data were collected in 2017 and 2016, respectively, the nutritional data collected did not reflect any product reformulation that may have taken place in the last few years. As a result, some products may have been misclassified according to healthfulness.

## Conclusion

This study has presented a unique perspective on the differences in exposure to food and beverage advertising among children and adolescents. Despite lower exposure among adolescents compared to children overall, both children and adolescents were exposed to unhealthy food categories and powerful marketing techniques. These data highlight the failure of current self-regulatory policies to protect children and the lack of consideration given to the protection of adolescents. Given the unique developmental vulnerabilities among both children and adolescents, government policies that protect all young people from the dangers of food and beverage advertising must be developed.

## Supplementary Information

**Additional file 1.** Numerator definition of food categories.

**Additional file 2.** Health Canada Nutrient Profile Model Thresholds.

## Data Availability

The data that support the findings of this study are available through license from Numerator and cannot be shared by the study authors. Numerator can be contacted regarding access to the data.

## Abbreviations

FLIP: Food Label Information Program
GRP: Gross rating points
CAI: Children’s Food and Beverage Advertising Initiative
NPM: Nutrient Profile Model
RA: Reference amount
