# Supplementary material for: Differences in child and adolescent exposure to unhealthy food and beverage advertising on television in a self-regulatory environment
Source: BMC Public Health. 2023 Mar 23;23:555. doi: 10.1186/s12889-023-15027-w (PMC10037770; doi:10.1186/s12889-023-15027-w)
Supplement: Supplementary file 1 — Additional file 1. Numerator definition of food categories. [file 12889_2023_15027_MOESM1_ESM.docx]

Numerator definition of food categories.

|  | **Condensed food category** | **Numerator food class** | **Definition** |
| --- | --- | --- | --- |
| 1 | **Bread** |  |  |
|  |  | Bakeries: Image | Manufacturers who primarily make baked goods. Image is typically branding where no specific product is mentioned |
|  |  | Bread; Rolls, Frozen Bread Dough | Bread and bread related products in fresh, refrigerated, and frozen form.*Exclude Crisp Breads, Flat Breads* |
| 2 | **Sweet baked goods/desserts** | |  |
|  |  | Cakes | Cakes and puddings. Exclude Frozen Treats |
|  |  | Cookies & Sweet Biscuits | Type: Butter cookies, chocolate finger cookies |
|  |  | Muffins & Donuts | Exclude all Muffin Mixes and English Muffins |
|  |  | Pies & Tarts, Snack Cake, Frozen, Unfrozen | Class includes all pies & tarts, as well as snack cakes.  These can be either frozen or ready to eat. |
|  |  | Sweet Rolls and Pastries: Frozen | This class contains only frozen & refrigerated pastries & sweet rolls. |
|  |  | Pudding and Flavoured Gelatin | Products suited to this class include wet packed ready to eat puddings & flavoured gelatins and dry-packaged pudding flavoured gelatin mixes.  Also included in this class are products labeled as both a pudding and pie filling.  Examples of brands to be included in the class are Jell-O and Hunts Snack Packs. |
|  |  | Ice Cream, Frozen Yogurt & Treats | This class will include ice cream, ice milk, sherbet, sorbet, frozen yogurt and frozen “novelty” treats.  All these brands are located together in the freezer section of the store.  They can be ice cream or fruit based with the addition of a variety of flavourings, syrups (e.g.: caramel), fruit, chocolate, nuts, marshmallows and candy (e.g.: Smarties, Rolo, Turtles).  Exclude Frozen Cakes and Pies, Refrigerated Yogurt |
|  |  | Pastries - Frozen or Refrigerated | Pastry is the name given to various kinds of baked goods made from ingredients such as flour, butter, shortening, baking powder or eggs. It may also refer to the dough from which such baked goods are made. Pastry dough is rolled out thinly and used as a base for baked goods. Common pastry dishes include pies, tarts, and quiches. Type: Pie Shells Frozen, Pastries Refrigerated |
| 3 | **Candy and chocolate** | |  |
|  |  | Candy without Chocolate | Candy, specifically sugar candy |
|  |  | Chocolate Bars, Candy with Chocolate | Exclude boxed chocolates |
|  |  | Chocolates: Boxed | Packaged chocolates, boxed or bagged |
|  |  | Confectionary Manufacturers Image | The term confectionery refers to food items that are rich in sugar. Confectionery items include sweets, lollipops, candy bars, chocolate, Cotton candy, and other sweet items of snack food. The term does not generally apply to cakes, biscuits, or puddings which require cutlery to consume. Images refers to branding with no specific product. |
| 4 | **Breakfast food** |  |  |
|  |  | Cereals: Cold | A cereal is a food product marketed to consumers as a breakfast food. Exclude infant cereals and rolled oats |
|  |  | Cereals: Hot | This class contains cereals which require some preparation prior to being eaten, either the addition of boiling water or cooking for a specified time. |
|  |  | Waffles, Pancakes, French Toast |  |
| 5 | **Dairy** |  |  |
|  |  | Cheese | Exclude Cottage Cheese |
|  |  | Dairy Products Image, Family Group, Assn | This class contains advertising by dairy companies as well as dairy producer councils, marketing boards and associations. Image refers to general branding with no specific product. Dairy Councils, Marketing Boards, Associations - advertising by national dairy groups is not broken out by the type of product shown. |
|  |  | Milk, Milk Powder, Milk Shakes | Milk is an opaque white liquid produced by the mammary glands of female mammals |
|  |  |  | Powdered milk is a manufactured dairy product made by evaporating milk to dryness. The purpose in drying milk is to preserve it. Exclude all Milk Flavourings, Evaporated Milk |
|  |  | Yogurt | This class will include all refrigerated yogurt in tubs, tubes and drink form. Exclude frozen yogurt |
| 6 | **Condiments** |  |  |
|  |  | Dips | Type:  Dip Mix, Spinach Dip, Roasted Red Pepper Dip. Excludes all gravy mixes & sauces |
|  |  | Honey | Exclude all products made with honey |
|  |  | Jams, Jellies & Marmalades | Exclude snack toppings, canned fruits |
|  |  | Sweet Spreads | Type:  Hazelnut spread, Honey butter |
|  |  | Table Syrup, Corn Syrup and Sundae Toppings | Type: Maple Syrup, Sundae Topping |
|  |  | Peanut Butter | Peanut butter is a food paste made primarily from ground-roasted peanuts, with or without added oil. |
| 7 | **Entrees and meat (including fish, poultry and meat products)** | | |
|  |  | Entrees | Type: Chicken Strips Frozen, Fish Sticks Frozen |
|  |  | Fish & Shellfish Canned | Canned Type: Tuna Canned, Pink Salmon Canned |
|  |  | Luncheon Meat | They can be bought pre-sliced in vacuum packs at a supermarket or grocery store, or they can be purchased at a delicatessen or deli counter, where they might be sliced to order. |
|  |  | Wieners & Franks | Hot dog wi^31^eners. |
|  |  | Pasta Meals - Package | Pasta (Italian for "dough") is a generic term for Italian variants of noodles, food made from a dough of flour, water and/or eggs, that is boiled. The word can also denote dishes in which pasta products are the primary ingredient, served with sauce or seasonings. Pasta meals come packaged with a dry sauce mix |
|  |  | Pizza, Frozen, Mixes | Type: Thin Crust Pizza, Pizza Rolls. Exclude all pizzas sold in restaurants |
| 8 | **Fruit and vegetables** | | |
|  |  | Fruit: Canned | This class only includes all fruits which come in cans, or fruit cups or in jars. Exclude all Dried, Fresh and Frozen Fruits |
|  |  | Fruit: Dried | Type : Raisins, Apricots. Exclude all Fresh, Canned or Frozen fruit |
|  |  | Fruit: Fresh | This class only includes all fruits which are fresh. Excludes all Dried, Canned & Frozen fruit |
|  |  | Fruit: Frozen | This class only includes all fruits which are frozen. Excludes all Fresh, Dried & canned fruit |
|  |  | Vegetables: Canned | Type: Diced Tomatoes, Vegetables Canned |
|  |  | Vegetables: Fresh | Exclude all frozen & canned & packaged vegetables |
|  |  | Vegetables: Frozen | Exclude all vegetables that are fresh, canned or dried |
|  |  | Vegetables: Packaged | Type: Beans Dried, Mashed Potatoes Package. Exclude all Fresh, Canned & Frozen vegetables |
| 9 | **Beverages (excluding milk and water)** | | |
|  |  | Juices, Drinks & Nectars | Products suited to this class include sweetened and unsweetened juice, drink and nectar beverages in liquid, frozen, and concentrate form.  Additionally dry-packaged mixes in powder and granule form that produce a beverage when mixed with water will be included. |
|  |  |  | This class will exclude the following products: |
|  |  |  | • Flavoured sparkling and/or flat waters (e.g.: Perrier flavoured with lemon or lime, DANONE Silhouette flavoured water) |
|  |  |  | • Iced tea and coffee drinks and mixes |
|  |  |  | • Flavoured Soft Drinks (e.g.: Diet Coke with Lime) |
|  |  |  | • Milk, Soy, & Rice based drinks (even when flavoured with fruits like strawberry) |
|  |  |  | • Sports Drinks (e.g.: Gatorade, Powerade) |
|  |  |  | • Extreme Energy Drinks (e.g.: Full Throttle, Red Bull) |
|  |  |  | • Cocktail Mixers (e.g.: Margarita, Pina Colada, Daiquiri, etc. mixers) |
|  |  |  | • Alcoholic Coolers (e.g.: Motts Clamato Caesar Cocktail Coolers) |
|  |  |  | • Juice and Drink beverages for babies and infants (e.g.: Heinz Baby Juices) |
|  |  | Milk Flavourings, Powder & Liquid | This class will include all liquid and dry powder or granular products to which milk or water is added to make a flavoured beverage.  These products may contain powdered milk, malt and or/cocoa as the base ingredients in addition to various sweetening and flavouring agents, most often chocolate or fruit like strawberry. |
|  |  | Soft Drink: Manufacturers & Associations | Type: Image, Association, Event Sponsor. Exclude all Juices & Drinks & Soft Drinks |
|  |  | Soft Drink: Diet | Exclude all regular Soft Drinks |
|  |  | Soft Drink: Regular | Soft drink is a beverage that does not contain alcohol. Exclude all Diet Soft Drinks |
|  |  | Sports Drinks | This class will exclude the following: Carbonated drinks, fruit drinks, extreme energy drinks |
|  |  | Extreme Energy Drinks | These brands are generally labelled “energy drinks”.  They are often herbal based and include one or more of the following ingredients – Ginseng, Guarana, Ginkgo Biloba & Taurine.  Sugar in the form of high fructose corn syrup, caffeine, vitamins (B6, B12) and minerals (calcium, iron) are often present as well. |
|  |  |  | Excludes Sports Drinks, Alcoholic Beverages |
| 10 | **Miscellaneous** |  |  |
|  |  | Food Manufacturers, Brokers, Exporters, Image & Family | Type: Importers, Manufacturers, Image. Exclude all food associations |
|  |  | Hot Snack & Sandwich Spreads & Toppings | This class contains all sandwich spreads, Toppings and Hot snacks. Exclude Cheese Spreads |
| 11 | **Snacks** |  |  |
|  |  | Portable Snacks | Products suited to this class include those whose function is to serve as a healthy type of snack food for both adults and children.  They are packaged to be portable as a component of a packaged lunch or to provide a light snack on the go between meals. This class will include all granola, cereal based, rice based, oat based, fruit based and muffin type snacks in bar, cluster, or square form.  Also included in this class are Fruit Snacks in both processed and pure form.  This class will exclude the following products: compartment snacks (e.g.: breadsticks with cheese dip, cookies with frosting) cookies & sweet biscuits cereals toaster pastries dried fruit, candy (e.g.: jujubes) |
|  |  | Snack Crackers, Soda Crackers | A cracker is a type of biscuit that is not sweet. |
|  |  | Snack Foods | Products suited to this class include all snack food items in chip, pretzel, stick, puffed, shoestring, popcorn and nut form whose main ingredient is potato, corn, rice, nuts, oats or a combination.  Additionally, RTE meat-based snacks including jerky type meats and meat snack sticks will be included. This class will exclude the following products: cookies, sweet biscuits & candy portable snack foods (e.g.: granola/cereal/cookie/protein bars, fruit snacks, Rice Krispie treats) toaster pastries compartment snacks (e.g.: cheese or dip with breadsticks, chips with dip) chip dips and salsa Lunch Kits & Mexican Kits with a snack food as an ingredient (e.g.: Lunchables, Lunchmates, Tortillas with cheese sauce)cheese based snacks stocked in the dairy case (i.e.: string cheese)snack & soda crackers |
|  |  | Compartment Snacks and Lunch Kits | Products suited to this class include pre-packaged snack type products comprised of two or more ingredients packaged in separate compartments.  The usual combinations are crackers/breadsticks together with a spread or dip such as cheese, peanut butter, etc.  Additionally, pre-packaged, refrigerated RTE compartmentalized meals intended to be used as lunch will be included. |
| 12 | **Water** |  |  |
|  |  | Water: Bottled | Exclude all Water Softeners & Additives |
| 13 | **Restaurants** |  |  |
|  |  | Fast food restaurants |  |
|  |  | Non-fast restaurants |  |
| Source: Numerator, 2019. | |  |  |
