# Supplementary material for: Differences in child and adolescent exposure to unhealthy food and beverage advertising on television in a self-regulatory environment
Source: BMC Public Health. 2023 Mar 23;23:555. doi: 10.1186/s12889-023-15027-w (PMC10037770; doi:10.1186/s12889-023-15027-w)
Supplement: Supplementary file 2 — Additional file 2. Health Canada Nutrient Profile Model Thresholds. [file 12889_2023_15027_MOESM2_ESM.docx]

Health Canada Nutrient Profile Model Thresholds.

|  | **Nutrient** | **Thresholds for foods** | **Thresholds for main dishes with a RA above 200g** |
| --- | --- | --- | --- |
| **Low in** | Saturated Fat | A total of 2 g SFA per RA or serving of stated size, whichever is the greater and ≤ 15% energy from the SFA | A total of 2 g SFA per 100g and ≤ 15% energy is from the SFA |
|  | Sodium | 140 mg per RA or serving of stated size whichever is the greater or 140 mg per 50 g of the product if the RA is ≤ 30g or 30 mL | 140 mg per 100g |
|  | Sugars | 5 g per RA or serving of stated size whichever is the greater or 5 g per 50 g of the product if the RA is ≤ 30g or 30 mL | 5 g per 100g |
| Source: Health Canada. Health Canada's Proposed Nutrient Profile Model for Restricting Marketing to Children. 2019. Unpublished [cited 2021 June 3]. | | | |
